# Diminishing the Pathogenesis of the Food-Borne Pathogen *Serratia marcescens* by Low Doses of Sodium Citrate

**DOI:** 10.3390/biology12040504

**Published:** 2023-03-26

**Authors:** Maan T. Khayat, Samar S. Elbaramawi, Shaimaa I. Nazeih, Martin K. Safo, El-Sayed Khafagy, Mohamed A. M. Ali, Hisham A. Abbas, Wael A. H. Hegazy, Noura M. Seleem

**Affiliations:** 1 Department of Pharmaceutical Chemistry, Faculty of Pharmacy, King Abdulaziz University, Jeddah 21589, Saudi Arabia; 2 Medicinal Chemistry Department, Faculty of Pharmacy, Zagazig University, Zagazig 44519, Egypt; 3 Department of Microbiology and Immunology, Faculty of Pharmacy, Zagazig University, Zagazig 44519, Egypt; 4 Department of Medicinal Chemistry, School of Pharmacy, Virginia Commonwealth University, Richmond, VA 23219, USA; 5 Department of Pharmaceutics, College of Pharmacy, Prince Sattam Bin Abdulaziz University, Al-kharj 11942, Saudi Arabia; 6 Department of Pharmaceutics and Industrial Pharmacy, Faculty of Pharmacy, Suez Canal University, Ismailia 41552, Egypt; 7 Department of Biology, College of Science, Imam Mohammad Ibn Saud Islamic University, Riyadh 11432, Saudi Arabia; 8 Department of Biochemistry, Faculty of Science, Ain Shams University, Cairo 11566, Egypt; 9 Pharmacy Program, Department of Pharmaceutical Sciences, Oman College of Health Sciences, Muscat 113, Oman

**Keywords:** *Serratia marcescens*, food bacterial contamination, natural anti-virulence agent, bacterial biofilms, sodium citrate, food preservation

## Abstract

**Simple Summary:**

Sodium citrate is a safe and frequently used food additive; the objective of this study is to assess the effectiveness of sodium citrate at low concentrations of 4% or 5% in combating *Serratia marcescens*, a food bacterial contaminant, by evaluating its anti-biofilm and anti-virulence properties. The current finding showed the significant ability of sodium citrate to inhibit the formation and virulence of bacterial biofilm. Furthermore, sodium citrate mitigated the bacterial pathogenesis on the infected mice. This study paves the way to use sodium citrate in low doses to reduce the harmful effects of *S. marcescens* and other bacteria that can cause foodborne illnesses. The use of sodium citrate could be an effective method to prevent the spread of this contaminating bacteria and improve food safety.

**Abstract:**

Protecting food from bacterial contamination is crucial for ensuring its safety and avoiding foodborne illness. *Serratia marcescens* is one of the food bacterial contaminants that can form biofilms and pigments that spoil the food product and could cause infections and illness to the consumer. Food preservation is essential to diminish such bacterial contaminants or at least reduce their pathogenesis; however, it should not affect food odor, taste, and consistency and must be safe. Sodium citrate is a well-known safe food additive and the current study aims to evaluate its anti-virulence and anti-biofilm activity at low concentrations against *S. marcescens*. The anti-virulence and antibiofilm activities of sodium citrate were evaluated phenotypically and genotypically. The results showed the significant effect of sodium citrate on decreasing the biofilm formation and other virulence factors, such as motility and the production of prodigiosin, protease, and hemolysins. This could be owed to its downregulating effect on the virulence-encoding genes. An in vivo investigation was conducted on mice and the histopathological examination of isolated tissues from the liver and kidney of mice confirmed the anti-virulence activity of sodium citrate. In addition, an in silico docking study was conducted to evaluate the sodium citrate binding ability to *S. marcescens* quorum sensing (QS) receptors that regulates its virulence. Sodium citrate showed a marked virtual ability to compete on QS proteins, which could explain sodium citrate’s anti-virulence effect. In conclusion, sodium citrate is a safe food additive and can be used at low concentrations to prevent contamination and biofilm formation by *S. marcescens* and other bacteria.

## 1. Introduction

*Serratia marcescens* is a Gram-negative bacterium that can be found in a variety of environments, including soil, water, and food [1,2]. It is often considered as an indicator of poor sanitation and hygiene practices. In food, it can be found in a variety of products, including raw and processed meats, dairy products, and fresh produce [3,4]. *S. marcescens* can cause food spoilage, discoloration, and off-flavors in food products. It can also cause foodborne illness if consumed in large enough quantities. The infectious dose of *S. marcescens* can vary depending on the susceptibility of the individual being infected. However, in general, it is believed that a relatively low number of *S. marcescens* can be enough to cause an infection in a healthy individual; it has been reported that the ingestion of as few as 10–100 *S. marcescens* can cause gastroenteritis in healthy individuals [5,6]. The symptoms of *S. marcescens* infection may include fever, chills, diarrhea, and abdominal cramps [3,7]. *S. marcescens* is an opportunistic pathogen that easily adapts to shifting physicochemical conditions, showing a considerable capability of surviving and thriving in diverse environments, such as disinfectant solutions, making it a major nosocomial pathogen which is often isolated from urinary tract, respiratory tract, soft tissue, ocular, and septic infections [1,8,9,10,11]. The *S. marcescens* adaptation and survival in diverse environments can be traced to a number of genetic factors, including chromosomally-encoded porins and efflux systems that contribute to the resistance to several antibiotics [2,12,13,14,15,16,17,18].

Bacterial biofilms are polymeric matrices of bacterial aggregates formed on both living and non-living surfaces that lead to a worsening of the bacterial infection and enhancing resistance to antibiotics [19,20]. Furthermore, the formation of biofilm by *S. marcescens* can lead to an increased risk of foodborne illness and food contamination leading to spoilage and discoloration and can affect the quality of food products [7,21,22]. *S. marcescens* produces a wide array of enzymes to establish its infection such as proteases, lipases, and hemolysins [11,23,24]. *S. marcescens* is known for its characteristic red pigment, prodigiosin, which can be produced in high quantities under certain conditions. Prodigiosin is linked to the virulence of *S. marcescens*, as it plays a role in the bacterium’s ability to cause disease, cytotoxicity, and antibiotic resistance [7,22,25,26].

Quorum sensing (QS) is the mechanism by which bacteria communicate with one another and regulate the production of virulence factors through an inducer-receptor process [27,28,29,30,31]. In Gram-negative bacteria, the primary inducers are acyl homoserine lactone (AHL). *Serratia* spp. employ two AHL-dependent QS systems, swrI/R [32] and smaI/R [33]. They utilize various AHLs, primarily C4 and C6 homoserine lactones, to regulate the expression of a range of virulence-related genes. These genes include prodigiosin, nuclease, lipase, protease, hemolysin, motility, and biofilm formation [15,22]. SmaR is Lux-type QS that senses its cognate inducers to form a receptor-inducer complex that binds the bacterial chromosome controlling the expression of virulence factors [15,22,34,35,36,37]. Hence, interfering with the QS system either through competition on the receptors or the quenching of AHL-mediation are considered very promising approaches to diminishing the bacterial virulence and easing their eradication [38,39,40,41,42,43,44,45,46]. This QS-targeting approach, unlike traditional antimicrobial agents, attenuates the bacterial virulence without influencing their growth resulting in almost nullifying the risk of resistance development [40,47,48,49,50,51,52,53].

Unfortunately, most anti-QS agents, such as gliptins, allopurinol, and adrenoreceptor blockers, and natural compounds, such as xylitol, ginerol, and vanilla extracts, have not been tried clinically, mostly due to a lack of extended studies on their toxicity and stability [28,40,54,55,56,57]. The screening of naturally safe, generally recognized as safe (GRAS), ingredients could be an efficient and applicable way to discover anti-QS candidates [21,41,55,58,59,60,61,62]. Sodium citrate is a safe food additive used as a flavor enhancer, helping to improve the taste and texture of food, such as processed cheese, gelatin mix, jams, ice cream, milk powder, sweets, carbonated beverages, and wine [63]. It is also used as an acidity regulator, emulsifier, and buffer, helping to maintain the stability of food and beverages during processing and storage [63]. Sodium citrate is considered to be safe in food by the FDA [63,64].

Medicinally, sodium citrate has been employed as an anticoagulant, and has also been used to treat metabolic acidosis and to prevent the development of renal stones [64,65]. Importantly, sodium citrate exhibits antimicrobial effects against *Streptococcus pneumoniae*, *S. mutans*, and other oral bacteria [66]. Furthermore, it has been reported that it possesses anti-QS properties against *Pseudomonas aeruginosa* [41], and efficient anti-biofilm activities [67,68].

Food safety is important for several reasons, the foremost being that it helps to prevent foodborne illnesses, which can cause a wide range of symptoms, from mild discomfort to serious and even life-threatening conditions. Another important aspect of food safety is that it helps to protect the reputation and financial stability of food businesses. In this context, the development of new, efficient, and safe antimicrobial candidates that do not affect food taste and consistence is a major priority in food safety. This study aimed to evaluate the anti-virulence activities of sodium citrate against one of the most considered food contaminants, *S. marcescens*. In vitro, in vivo, and in silico studies were conducted to evaluate sodium citrate anti-virulence activities.

## 2. Materials and Methods

### 2.1. Bacteria, Media, and Growth Conditions

*S. marcescens* used in this study was obtained from culture collection of the Microbiology and Immunology Department, Faculty of Pharmacy, Zagaig University, Egypt [21,69,70]. The microbiological media were obtained from Oxoid (Hampshire, UK). Sodium citrate and other chemicals were obtained from Sigma-Aldrich (St. Louis, MO, USA). *S. marcescens* overnight fresh cultures were prepared in Luria–Bertani (LB) broth and adjusted to optical density equal to 0.5 McFarland Standard 1 × 10^8^ cfu/mL (O.D600 of 0.4), prior to being used in each experiment.

### 2.2. Effect of Sodium Citrate on Bacterial Growth

To exclude the possibility of sodium citrate anti-virulence due to affecting bacteria growth, low sodium citrate concentrations of 4% and 5% were used for the study [41]. Furthermore, the optical densities (600 nm) and viable counts of *S. marcescens* cultures in Luria–Bertani (LB) broth with and without sodium citrate of 4% or 5% were measured and compared [23,35].

### 2.3. Effect of Sodium Citrate on Biofilm Formation

Crystal violet method was employed to assess the effect of sodium citrate at 4% and 5% on biofilm formation as described previously [50,71]. *S. marcescens* strain is considered as a strong biofilm-forming bacterium [23,69]. Briefly, a bacterial suspension was prepared in Tryptic Soy broth (TSB) (OD_600_ of 0.4). Aliquots (10 μL) of the bacterial suspensions were added to 1 mL amounts of fresh TSB with or without sodium citrate (4% and 5%). Then, 100 μL of TSB (with or without sodium citrate) were transferred into microtiter plates and incubated overnight at 37 °C. After aspiration of the planktonic cells, the biofilm forming cells were fixed with methanol for 20 min and stained with crystal violet (1%) for 25 min. After twenty minutes, the excess crystal violet was washed out and the adhered crystal violet was extracted by glacial acetic acid (33%). The absorbances were measured at 590 nm. The non-cultured TSB was used as negative control, while the TSB cultured with untreated *Serratia* was positive control. The absorbances of *Serratia* treated with sodium citrate were measured and evaluated as percentage change from untreated *Serratia* control using formula: [(OD_590_ untreated control—OD_590_ citrate treated)/OD590 untreated control] × 100.

### 2.4. Effect of Sodium Citrate on Motility

The effect of sodium citrate at 4% and 5% on bacterial swarming motility was performed as described previously [72,73]. Briefly, 5 µL of optically adjusted bacterial suspensions were centrally spotted on the surface of Mueller–Hinton (MH) agar with or without 4% or 5% sodium citrate. After overnight incubation, the swarming zones were measured. The diameters of *Serratia* treated with sodium citrate were measured in mm and calculated as percentage change from untreated *Serratia* control using formula: [(Untreated control swarming zone − Citrate treated swarming zone)/Untreated control swarming zone] × 100.

### 2.5. Anti-Proteolytic Activity Assay

Skim milk method was used to evaluate the effect of sodium citrate (4% or 5%) on the protease activity [40,58]. The supernatants were collected by centrifugation of LB cultures of optically adjusted bacterial growth in the presence or absence of sodium citrate 4% or 5%. Skim milk LB agar plates were prepared by adding 5% skim milk, not containing any proteases. The preformed wells in the skim milk agar were filled with supernatants containing protease. The diameters of the clear zones that indicate proteolytic activities were measured after overnight incubation. Sterilized LB was poured into wells as negative control, while untreated Serratia protease was considered as positive control [15,21,23,70]. The clear zone formed around the sodium citrate treated *Serratia* were calculated as percent change from clear zones formed around wells contain untreated *Serratia* control.

### 2.6. Effect of Sodium Citrate on the Production of Prodigiosin

The quantification of the produced prodigiosin by *S*. *marcescens* was performed in the presence or absence of 4% or 5% sodium citrate as described previously [15,21,23]. The OD of *Serratia* suspensions were adjusted to OD600 of 0.4 (1 × 10^8^ CFU/mL) and overnight inoculated in fresh LB broth (2 mL) provided or not with sodium citrate at 28 °C. After centrifugation, the prodigiosin was extracted from supernatants by ethanol provided with 4% 1 M HCl. The absorbances of extracted prodigiosin were measured at 534 nm and the degree of inhibition was calculated as percentage change from untreated *Serratia* control.

### 2.7. Effect of Citrate on the Expression of Virulence-Encoding Genes

The RNA was obtained from *S. marcescens* cultures grown in the presence or absence of 5% citrate using Purification Kit Gene JET RNA (Thermo Scientific, Waltham, MA, USA) and kept at −80 °C as described [59,74]. A quantitative real-time PCR was conducted to quantify the expression of genes involved in the *S. marcescens* virulence. The primers used are listed in Table 1. The expression levels were normalized to the housekeeping gene *rpl*U. A high-capacity cDNA reverse transcriptase kit (Applied Biosystem, Waltham, MA, USA) was employed to synthesize the cDNA, and the cDNA was amplified using the SYBR Green I PCR Master Kit (Fermentas, Waltham, MA, USA) in a Step One instrument (Applied Biosystem, Waltham, MA, USA). A melting curve was performed according to the instructions of the manufacturer, and the relative expressions were calculated using the comparative threshold cycle (∆∆Ct) method [75].

### 2.8. Histopathological Evaluation of Sodium Citrate Effect on the Liver and Kidney Tissues of the Infected Mice

Twenty albino mice (three-week old) were allowed to adapt in optimal housing conditions, and then divided into four groups each comprising five mice. The first group, which was the test group, was intraperitonially injected with *S. marcescens* treated with 5% sodium citrate. The second group, which was the positive control, was injected with untreated *S. marcescens*. The third group was not treated, while the fourth group was treated with sterile PBS as negative controls. After five-day observation, the mice were euthanized by cervical dislocation. Liver and kidneys were dissected from mice, washed with normal saline, and fixed in 10% formalin for histopathological inspection. After the dehydration in ethyl alcohol increasing concentrations, the samples were cleared in xylol, impregnated, and embedded in paraffin wax. Rotatory microtome was used to cut 5 μm thick sections, then stained with hematoxylin and eosin (H and E × 200) stain and examined using a Leica DM750 HD digital microscope (Mannheim, Germany) [50,76].

### 2.9. In Silico Molecular Docking Study

Sodium citrate chemical structure was obtained from PubChem database (https://pubchem.ncbi.nlm.nih.gov/ accessed on 14 October 2022) as canonical SMILES. The structure of citrate was prepared through energy minimization using 0.1 Kcal/mol/Å^2^ gradient RMS on Molecular Operating Environment (MOE 2019.012). Docking method was performed through Alpha triangle placement with Amber10: EHT forcefield. Triangle matcher is the docking placement methodology, where London dG is the initial scoring function and GBVI/WSAdG is the final scoring function.

*S. marcescens* QS transcriptional regulator SmaR (Uniprot Entry: Q14RS3, www.uniprot.org accessed on 28 May 2022) has no resolved crystal structure [38], so a SWISS MODEL (https://www.expasy.org/resources/swiss-model; accessed on the 28 May 2022) was used as reported [15,77,78,79,80,81]. *Chromobacterium violaceum* CviR transcriptional regulator (PDB: 3QP5, 3.25 Å) utilized as a template for construction of *S. marcescens* SmaR model. *C. violaceum* CviR transcriptional regulator is the top-ranked template according to the sequence identity from seven recommended templates. The active site in the SWISS model was determined by the MOE site finder module which matched the co-crystallized ligand (HLC) binding site of the template.

### 2.10. Statistical Evaluation

The experiments were conducted in triplicates and the results were averaged. The results were expressed as mean ± standard, and the One-way ANOVA test was used to attest the statistical significance, and significance was considered when *p* < 0.05.

## 3. Results

### 3.1. Sodium Citrate at 4% or 5% Has No Effect on the Bacterial Growth

**Figure 1:**
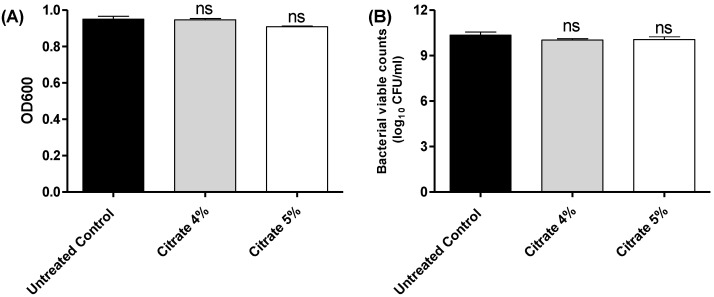
The effect of sodium citrate at 4% or 5% on the *S. marcescens* growth. There was no significant effect of citrate at tested concentrations on (**A**) the optical densities of bacterial growth, (**B**) bacterial counts. One-way ANOVA test was employed to assess significance; ns, non-significant: *p* > 0.05.

Sodium citrate showed no significant effect on bacterial growth (*p* > 0.05) (Figure 1).

### 3.2. Sodium Citrate Diminishes the Formation of Biofilms

**Figure 2:**
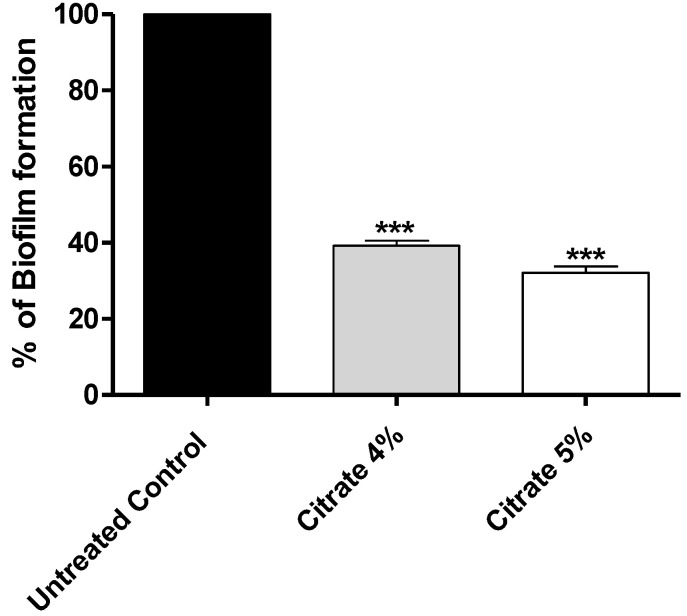
The effect of sodium citrate at 4% or 5% on the biofilm formation. Citrate at 4% or 5% significantly diminished the biofilm formation; the inhibition percentages were 61% and 68%, respectively. The data are presented as percentage change from control, and One-way ANOVA test was employed to assess significance; ***: *p* ≤ 0.001.

Sodium citrate at 4% or 5% significantly diminished the biofilm formation with percentages exceeding 60% when compared with the untreated control. The data are presented as percentage change from the untreated control (Figure 2).

### 3.3. Sodium Citrate Diminishes the Swarming Motility of S. marcescens

**Figure 3:**
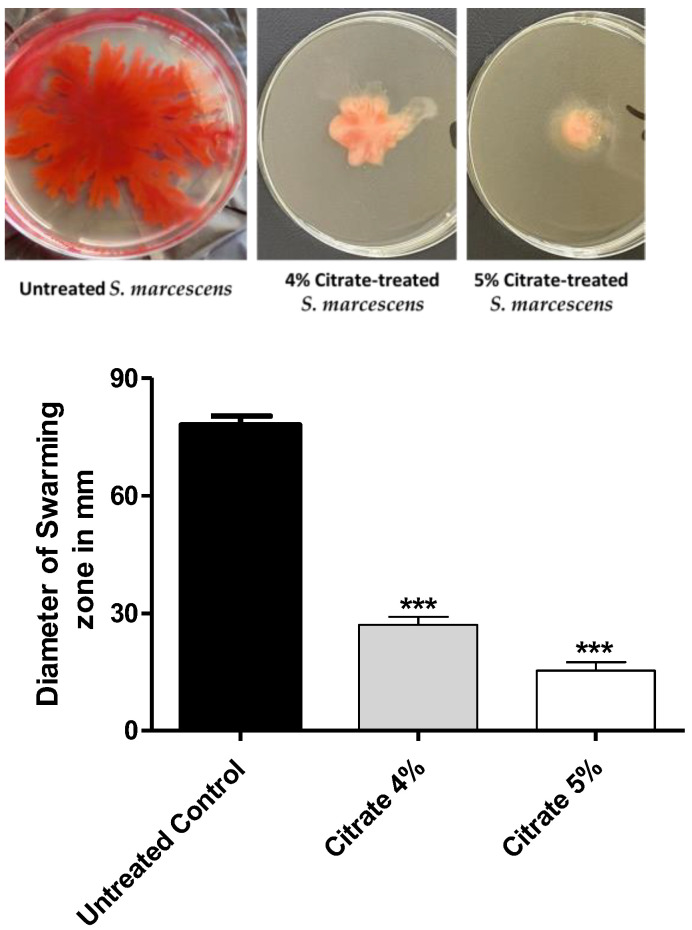
The effect of sodium citrate at 4% or 5% on the *S. marcescens* swarming motility. Citrate at 4% or 5% significantly decreased the swarming zones of *S. marcescens* by about 62% or 78%, respectively. The One-way ANOVA test was employed to assess significance; ***: *p* ≤ 0.001.

The influence of citrate on the *S. marcescens* swarming motility was assessed. Citrate significantly decreased the swarming zone at the tested concentrations. Citrate at concentrations 4% or 5% decreased the bacterial motility by 62% or 78%, respectively (Figure 3).

### 3.4. Sodium Citrate Decreases the Activity of Protease

**Figure 4:**
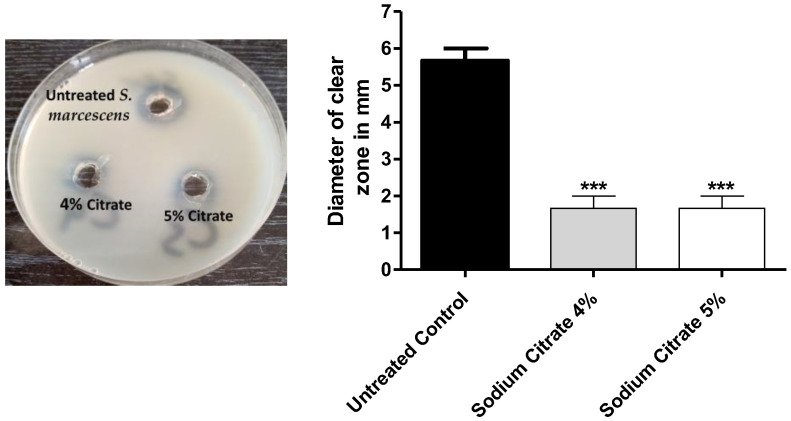
The effect of sodium citrate at 4% or 5% on the protease production. Citrate at 4% or 5% significantly decreased the clear zones around the wells loaded with extracellular protease (60%), indicating decrease in protease production. The One-way ANOVA test was employed to assess significance; ***: *p* ≤ 0.001.

Citrate significantly decreased the clear zones around preformed wells loaded with cell-free supernatants containing extracellular protease, indicating a significant decrease in its activity (*p* < 0.001). The decrease percentages in the protease production were about 60% (Figure 4).

### 3.5. Sodium Citrate Decreases the Production of Prodigiosin

**Figure 5:**
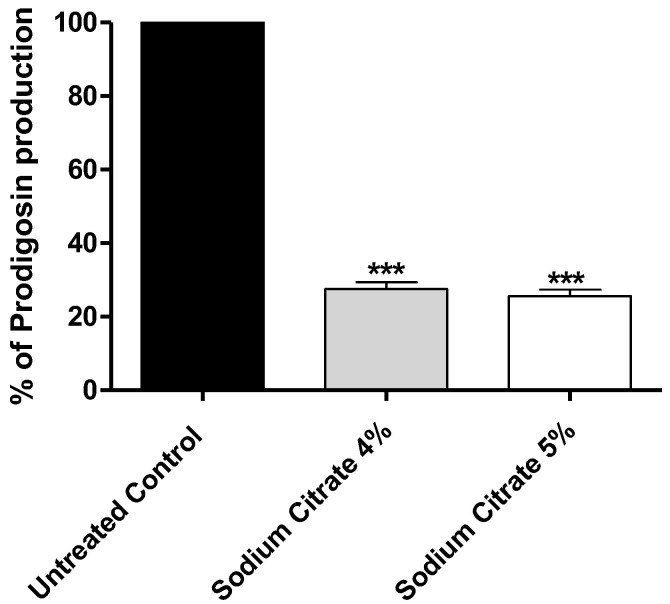
The effect of sodium citrate at 4% or 5% on production of prodigiosin. Citrate at 4% or 5% significantly diminished production of prodigiosin; the inhibition percentages were about 70%. The data are presented as percentage change from control, and One-way ANOVA test was employed to assess significance; ***: *p* ≤ 0.001.

Citrate significantly decreased the production of prodigiosin by more than 70% (Figure 5).

### 3.6. Sodium Citrate Downregulates the Expression of Virulence Genes

**Figure 6:**
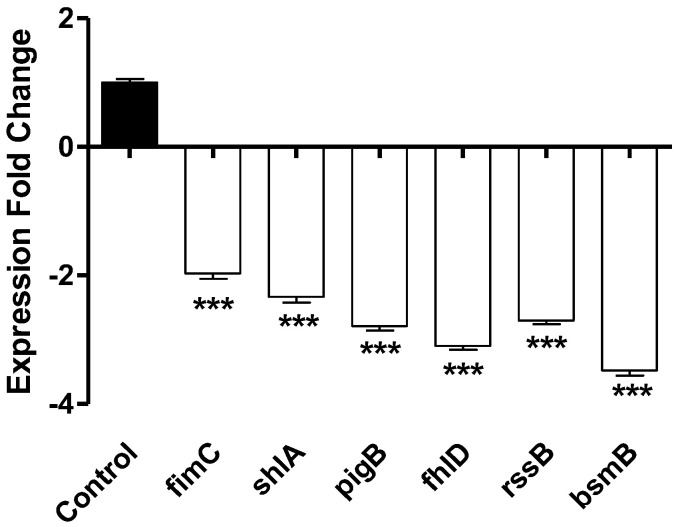
The effect of sodium citrate at 5% expression of virulence genes in *S. marcescens*. Citrate at 5% significantly decreased the expression of the virulence encoding genes The One−way ANOVA test was employed to assess significance; ***: *p* ≤ 0.001.

Sodium citrate (5%) significantly downregulated the expression of all the tested genes from two to four folds (Figure 6).

### 3.7. Sodium Citrate Alleviates the Histopathological Changes in Liver and Renal

**Figure 7:**
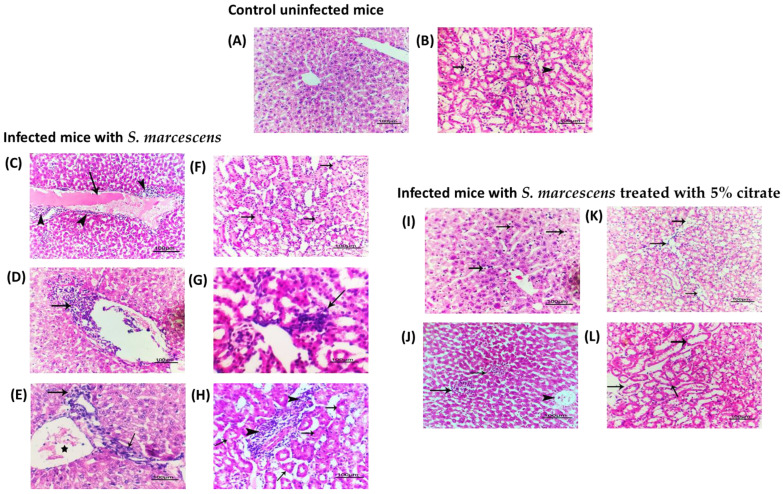
Histopathological investigation of liver and renal tissues. The liver and kidney tissues were isolated from mice groups that were either kept uninfected (negative control), injected with untreated *S. marcescens* (positive group), or injected with *S. marcescens* treated with 5% sodium citrate. (**A**) Photomicrograph of H and E-stained liver section of negative control group (uninfected mice) showing normal tissue architecture and cellular details. (**B**) Photomicrograph of H and E-stained kidney section of control group showing normal renal cortex with normal glomeruli (arrows) and renal tubules (arrowhead). Photomicrograph of H and E-stained liver sections of infected group with untreated *S. marcescens* showing: (**C**) severe congestion of hepatic blood vessels (arrow) with perivascular inflammatory cells infiltration (arrows head), (**D**) massive margination and diapedesis of inflammatory cells (arrow), and (**E**) peri ductal inflammatory cells infiltration (star) with giant cells (arrows). Photomicrograph of H and E-stained kidney sections of infected group with untreated *S. marcescens* showing: (**F**) atrophy and degeneration of some renal tubules (arrows), (**G**) focal area of leucocytic cells infiltration (arrows), and (**H**) perivascular inflammatory cells infiltration (arrows head) with individualization and atrophy of some renal tubules (arrows). Photomicrograph of H and E-stained liver sections of mice group injected with 5% citrate-treated *S. marcescens* showing: (**I**) normal tissue architecture with diffuse von Kupffer cells infiltration (arrows), and (**J**) mild congestion of hepatic blood vessels (arrowhead) with mild inflammatory focal cells infiltration. Photomicrograph of H and E-stained kidney sections of mice group injected with 5% citrate-treated *S. marcescens* showing: (**K**) minimal focal areas of cystic dilation of some renal tubules (arrows), and (**L**) mostly normal renal medulla with mild focal individualization of some renal tubules (arrows). (Scale bar =100 µm).

To determine the effect of sodium citrate (5%) on abrogating the *S. marcescens*-induced pathogenesis in the liver and kidney tissues of infected mice; demonstrative photomicrographs were captured from the infected mice with sodium citrate-treated or -untreated *S. marcescens* (Figure 7). The kidney or liver of mice from the uninfected group or sterile PBS-injected group showed normal tissue architecture and cellular details (Figure 7A,B). The liver tissues from the mice group injected with untreated *S. marcescens* showed severe congestion of hepatic blood vessels with perivascular inflammatory cells infiltration (Figure 7C–E). Similarly, the renal tissues from the mice group injected with untreated *S. marcescens* showed atrophy and degeneration of some renal tubules with focal areas of leucocytic cells infiltration (Figure 7F–H). On the other hand, the tissues isolated from the mice group injected with sodium citrate-treated *S. marcescens* showed only mild congestion of hepatic blood vessels (Figure 7I,J) and minimal focal areas of cystic dilation of some renal tubules (Figure 7K,L). The present findings reveal the marked in vivo diminishing effect of citrate on *S. marcescens* pathogenesis.

### 3.8. In Silico Competition on QS Targets

**Figure 8:**
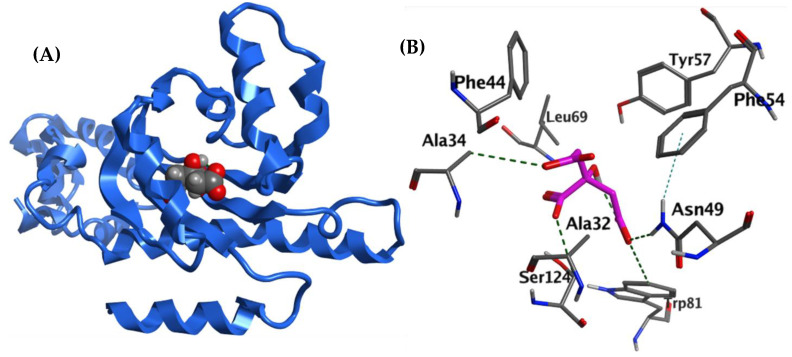
(**A**) 3D Citrate (Filled space shape)—*S. marcescens* SmaR model (blue ribbon). (**B**) 3D citrate—*S. marcescens* SmaR interactions, citrate is thick purple sticks, amino acid residues are grey sticks, and H-bonds are shown as dark green dots.

In a comparison of citrate with a natural ligand, HLC: [4-(4-chlorophenoxy)-N-[(3S)-2-oxooxolan-3-yl]butanamide], sodium citrate could bind and block ethe *S. marcescens* QS SmaR receptor (docking energy: −5.0162 Kcal/mol compared with −6.9484 Kcal/mol for the HLC), Table 2. Citrate and HLC appear to form several conserved hydrophobic interactions with the protein, including Ala34, Phe44, Tyr57, and Trp81, in addition to other unique hydrophobic interactions by these two ligands (Table 2). Importantly, while citrate formed several H-bond interactions with Ala34, Asn49, Trp81, and Ser124, HLC only made one hydrogen bond interaction with Trp53. Figure 8 shows the interacting amino acids present in the active site of *S. marcescens* SmaR, proposing that citrate may be an effective QS inhibitor.

## 4. Discussion

Bacterial contamination in food can occur at any point during food processing, from growing and harvesting to packaging and distribution. Contamination can come from a variety of sources, such as water, soil, animals, and even human handlers. *Serratia* can cause food contamination through a variety of means. It can be present in raw materials used in food production, such as fruits, vegetables, and meat. It can also contaminate food processing equipment and surfaces, and can survive on these surfaces for long periods of time [82,83]. Once established, *Serratia*, a well-known food pathogen, can form biofilms on food and surfaces, which can make the bacteria more resistant to cleaning and disinfection [84,85]. Critically, *Serratia* can cause infections in humans, particularly in individuals with compromised immune systems [2,10,86]. Anti-virulence agents are compounds that target specific virulence factors in bacteria, rather than targeting the bacteria themselves [39,87]. This approach is seen as a way to combat bacterial infections without promoting antibiotic resistance, as the bacteria are not killed but are instead rendered less capable to cause harm [70,73,88]. Examples of anti-virulence agents include compounds that target bacterial QS or biofilm formation [30,38,47]. There are diverse chemical compounds and drugs that have shown considerable anti-virulence and anti-QS activities [19,20]; however, their clinical application requires extended toxicological and pharmaceutical studies [49,71]. Additionally, competing against the bacterial contamination and biofilms in food requires agents that do not influence the taste, odor, or consistency. This can be achieved by using already safe food ingredients that confers anti-biofilm, anti-virulence, and anti-QS activities [21,41,55,58,60,61,89,90,91].

Sodium citrate is a common safe food additive that is used in diverse foods and drinks, beside its uses in medicine [41,64,65,92]. The current study aimed to evaluate the effect of sodium citrate on bacterial virulence at very low concentrations (4% or 5%) that can be used to protect food preparations from bacterial contaminants, such as *S. marcescens*. Targeting bacterial virulence is a promising approach that does not stress bacteria to develop resistance [48,93,94]. Prior to assessing the anti-virulence activities of sodium citrate, its effect on bacterial growth at concentrations of 4% or 5% was determined. Sodium citrate at tested concentrations did not show any significant influence on bacterial growth indicating that any effect of virulence is owed solely to citrate’s anti-virulence activity and not due to its effect on growth.

Bacterial virulence factors, in particular biofilm formation, are an additional burden that enhance the resistance of and complicate bacterial infections [95,96,97,98]. The contamination of food processing equipment, storage containers, and of food itself mean biofilms can lead to economic loss, and it can infect consumers leading to clinical symptoms and illness [96,99]. Sodium citrate significantly diminished biofilm formation by *S. marcescens*. The biofilm formation is a multistep process starting with the adhesion mediated by different types of pili [29,96,100,101]. Importantly, *S. marcescens* depends on type I pili encoded by the operon *fimABCD* to initiate biofilm formation [102]. The transcriptional factors BsmA/B enhance the production of *S. marcescens* type I pilus [36]. In agreement with phenotypic findings in which sodium citrate decreased the biofilm formation, sodium citrate at 5% significantly decreased the expression of the fimbrial protein-encoding gene *fimC* and type I fimbriae regulatory protein *bsmB* gene (Figure 6, Table 1).

Additionally, it was demonstrated that there is a correlation between bacterial motility and flagellar expression with the formation of biofilms. Bacterial strains that exhibit motility tend to have a robust biofilm formation, whereas non-motile mutants have weaker biofilm formation [73,103]. Along with the anti-biofilm activity, sodium citrate significantly decreased *S. marcescens*’ swarming motility. *S. marcescens* employs RssAB, a two-component system, to control swarming [104,105]. The *S. marcescens* swarming control is multifactorial by nutrient status, temperature, and mainly the flagellar regulatory master operon flhDC, in addition to the complex regulatory network encoded by the *rsmA* gene [24,106]. The flhDC operon is composed of *flhC* and *flhD* genes that encode flagellar transcriptional regulator FlhC and flagellar transcriptional activator FlhD [11,25]. In agreement with diminishing swarming motility, sodium citrate down regulated the genes involved in motility *flhD* and *rssB*.

Virulent bacteria employ an arsenal of extracellular enzymes to establish their infection into the host, such as protease, hemolysins, lipases, and other enzymes [52]. Furthermore, some enzymes, such as protease and lipases, could acquire a potential deteriorative effect when bacteria contaminates food [84]. Sodium citrate decreased the production of protease significantly by *S. marcescens*. *S. marcescens* can penetrate the host epithelial barriers by destroying the tissues using pore-forming toxins such as ShlA. The ShlA toxin is a two-component secretion system that recruits the Ca^2+^ influx triggering mechanism in the host cells [23,35]. The current results showed the significant ability of sodium citrate to downregulate the expression of the pore-forming toxin-encoding gene *shlA*. In addition to extracellular enzymes, *S. marcescens* produce its significant red pigment prodigiosin under the control of QS by the biosynthetic operon, pigA-N [22,26,83]. Although prodigiosin has several biomedical and industrial uses including algicidal, anticancer, insecticidal, bactericidal, antiprotozoal, antimalarial, immunosuppressive agents, and colorants, it plays essential roles in the *Serratia* virulence, such as in antibiotic resistance, the modulation of immunity, and causing diseases [7,26]. Furthermore, this reddish pigment is an indicator of food contamination and unsuitability to be consumed [7]. Sodium citrate significantly decreased the production of prodigiosin and also significantly downregulated its encoding gene *pigB*.

As detailed previously, since sodium citrate at a concentration of 5% has no influence on the bacterial growth, the in vivo evaluation of *S. marcescens* was conducted using sodium citrate at 5% by the intraperitoneal injection of sodium citrate-treated or -untreated *S. marcescens*. The liver and kidney tissues were isolated for histopathological examination. The tissues isolated from mice injected with untreated *S. marcescens* showed signs of severe inflammation, congestion, and the infiltration of white blood cells. On the other hand, the tissues isolated from mice injected with *S. marcescens* treated with sodium citrate showed marked relief of all signs of inflammation and congestion indicating the significant sodium citrate diminishing effect on pathogenesis.

QS is the main regulator of bacterial virulence that controls the expression of a wide diversity of virulence factors. In Gram-negative, the Lux-type QS receptors are the predominant, such as the *S. marcescens* SmaR QS receptor [21,22,23,107]. The data obtained from a virtual docking study of sodium citrate into a SmaR QS receptor suggest citrate can potentially bind with the receptor and modulate its activity. These findings could explain the anti-QS activity of sodium citrate, which could account for its anti-virulence activity. It is worth mentioning that sodium citrate showed considerable anti-QS activities against *Pseudomonas aeruginosa* QS receptors, in which sodium citrate down regulated the QS encoding genes and interferes with Lux-type QS receptors [41]; this is in agreement with the current results.

## 5. Conclusions

Sodium citrate is a known, safe, economical food additive and is widely used in food manufacturing. Food contamination by bacteria is hazardous and could lead to infections and economic losses. The anti-virulence activity of sodium citrate was evaluated against one of the considered food contaminants *S. marcescens*. Sodium citrate at concentrations of 4% or 5% significantly decreased the bacterial biofilm formation, motility, and production of protease and prodigiosin pigment. Consistent with the in vitro results, sodium citrate decreased the expression of virulence encoding genes and showed obvious in vivo protection of mice from the *S. marcescens* pathogenesis. The anti-virulence activity of sodium citrate could be due to its binding affinity to QS receptors. These findings elaborate on the possibility of using sodium citrate at 4% or 5% to diminish bacterial biofilms and virulence, especially since it can be used at low concentrations that do not affect food taste, odor, or consistency.

## Figures and Tables

**Table 1:** The primers used in this study.

Gene	Sequence (5′–3′)	Gene Significance	Reference
*fim*C	For: AAGATCGCACCGTACAAACC Rev: TTTGCACCGCATAGTTCAAG	Fimbria (adhesion)	[23,54]
*flh*D	For: TGTCGGGATGGGGAATATGG Rev: CGATAGCTCTTGCAGTAAATGG	Flagella (motility)	[23,54]
*bsm*B	For: CCGCCTGCAAGAAAGAACTT Rev: AGAGATCGACGGTCAGTTCC	Type I pilus (adhesion)	[23,54]
*pig*B	For: GAACATGTTGGCAATGAAAA Rev: ATGTAACCCAGGAATTGCAC	Motility	[23,54]
*rss*B	For: TAACGAACTGCTGATGCTGT Rev: GATCTTGCGCCGTAAATTAT	Motility	[23,54]
*shl*A	For: GCGGCGATAACTATCAAAAT Rev: ATTGCCAGGAGTAGAACCAG	Pore forming (hemolysis)	[23,54]
*rpl*U	For: GCTTGGAAAAGCTGGACATC Rev: TACGGTGGTGTTTACGACGA	Housekeeping gene	[23,54]

**Table 2:** Docking results for both citrate and HLC with *S. marcescens* SmaR.

Ligand	S Score (Kcal/mol)	H-Bond Interactions	Hydrophobic Interactions
Citrate	−5.0162	Ala34, Asn49, Trp81, Ser124	Ala32, Phe44, Asn49, Tyr57, Leu69, Trp81
HLC	−6.9484	Trp53	Ala32, Phe44, Tyr57, Trp81, Ile105, Val122, Ser124

## Data Availability

Not applicable.
